# Epithéliopathie rétinienne diffuse

**DOI:** 10.11604/pamj.2017.28.182.11355

**Published:** 2017-10-27

**Authors:** Yassine Abaloun, Abdelhadi Omari

**Affiliations:** 1 Université Mohammed V Souissi, Service d’Ophtalmologie de l’Hôpital Militaire Mohamed V, Rabat, Maroc

**Keywords:** Epitheliopathie rétinienne diffuse, ERD, CRSC chronique, Diffuse retinal epitheliopathy, DRPE, chronic central serous retinopathy (CSR)

## Abstract

We report the case of a 52-year old man with no previous significant medical history presenting with progressive decrease in visual acuity (VA) of the right eye evolving over 10 years. Corrected visual acuity was 2/10 - P6 in the RE and 10/10 - P2 in the LE. The examination of the anterior segment was unremarkable. Fundus examination showed alteration of the pigment epithelium (APE) in the RE associated with osteoblast-like pigment migrations involving the macula and a wide area due to gravitational descent of the superior temporal arcade onto the lower temporal quadrant. The left eye had a similar appearance especially in the inter-papillo-macular region (A,B). Fluorescein angiography showed early hyperfluorescence areas in the PE depigmented areas associated with pigment migrations giving a comet tail appearance by gravity casting in both eyes (C,D). Optical coherence tomography (OCT) showed retrofoveolar epithelial detachment (PED) at the level of the RE (E). The patient received Diamox therapy with regular monitoring to manage possible leakage points. Patient ’s evolution was marked by PED regression and VA improvement.

## Image en médecine

Nous rapportons le cas d’un homme de 52 ans sans ATCD consultant pour une baisse progressive de l’acuité visuelle de l’œil droit évoluant depuis 10 ans. L’acuité visuelle corrigée est de 2/10 - P6 à l’OD et de 10/10 - P2 à l’OG. L’examen du segment antérieur est sans particularité. Le FO retrouve au niveau de l’OD une altération de l’épithélium pigmentaire (AEP) associée à des migrations pigmentaires d’aspect ostéoblastique intéressant la macula et une zone étendue par coulée gravitationnelle de l’arcade temporale supérieure jusqu’au quadrant temporal inférieur. Au niveau de l’œil gauche on note un aspect similaire surtout dans la région inter papillo-maculaire (A,B). L’angiographie à la fluorescéine montre dans les 2 yeux une hyperfluorescence précoce des zones de dépigmentation de l’EP associé à des migrations pigmentaires donnant un aspect de queue de comète par coulée gravitationnelle (C,D). L’OCT montre un DEP retro-fovéolaire au niveau de l’OD (E). Le patient a été mis sous Diamox avec une surveillance régulière pour prendre en charge l’éventuelle apparition de points de fuite .L’évolution s’est faite vers une régression du DEP et une amélioration de l’AV.

**Figure 1:**
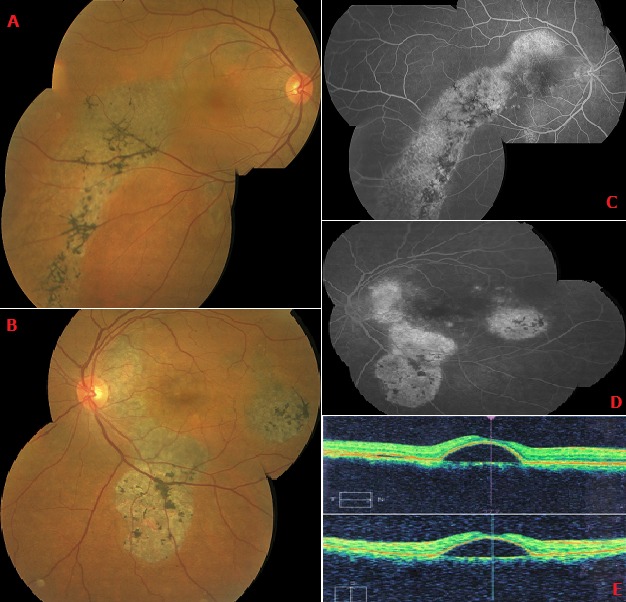
Photos du fond d’œil (A: œil droit, B: œil gauche) montrant un remaniement de l’EP avec des migrations pigmentaires d’aspect osteoblastique. Angiographie rétinienne à la fluorescéine aux temps tardifs: (C: œil droit, D: œil gauche) montrant une hyperfluorescence hétérogène des zones d’AEP donnant un aspect en queue de comète, (E) OCT maculaire de l’OD montrant un DEP retrofovéolaire

